# Cheap, Healthy Houses

**Published:** 1871-07

**Authors:** 


### CHEAP, HEALTHY HOUSES.

A TWO-STORY house can be made of brick by building the wall hollow, requiring one third less than if solid, with the advantage that the hollow wall may be made as substantial and durable, and at the same time drier, cooler in summer and warmer in winter, a great deal, than if solid; because between the bricks there is a stratum of air which naturally gives the advantages named. The attention of builders, and of all who contemplate building for themselves, is specially called to these suggestions, for it is always of the utmost importance that the houses we live in should be as dry as possible; warm in winter and cool in summer.

